# (22*E*,24*R*)-3α,5-Cyclo-5α-ergosta-22-en-6-one

**DOI:** 10.1107/S1600536810052906

**Published:** 2011-01-08

**Authors:** Liu-qing Sheng, Fang Zeng, Fei Chen, Chun-nian Xia

**Affiliations:** a Department of Pharmaceutical Science, Jinhua Polytechnic, Jinhua 321007, People’s Republic of China; b College of Pharmaceutical Science, Zhejiang University of Technology, Hangzhou 310032, People’s Republic of China

## Abstract

In the title mol­ecule, C_28_H_44_O, the two six-membered rings have a chair conformation and the two five-membered rings haveenvelope conformations. The crystal packing exhibits no short inter­molecular contacts. The absolute configuration was assigned to correspond with that of the known chiral centres in a precursor molecule, which remained unchanged during the synthesis of the title compound.

## Related literature

Many analogues of brassinolide as plant regulators have been isolated from a wide variety of plants, and many attempts have been undertaken to synthesize these brassinosteroids, see, for example: Aburatani *et al.* (1987 ▶); Brosa *et al.* (2001 ▶); Brosa & Santamarta (1999 ▶); McMorris *et al.* (1993 ▶); Clouse (1996 ▶, 2002 ▶). For related structures, see: Chen *et al.* (2009 ▶); Xia *et al.* (2005 ▶).
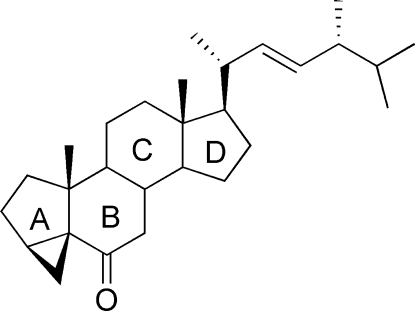


## Experimental

### 

#### Crystal data

C_28_H_44_O
*M* *_r_* = 396.63
Orthorhombic, 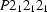

*a* = 7.6628 (19) Å
*b* = 10.516 (3) Å
*c* = 29.855 (8) Å
*V* = 2405.9 (11) Å^3^
*Z* = 4
Mo *K*α radiation
μ = 0.06 mm^−1^
*T* = 123 K
0.42 × 0.36 × 0.34 mm

#### Data collection

Rigaku AFC10/Saturn724+ diffractometer
18847 measured reflections
3143 independent reflections
2973 reflections with *I* > 2σ(*I*)
*R*_int_ = 0.028

#### Refinement

*R*[*F* ^2^ > 2σ(*F* ^2^)] = 0.038
*wR*(*F* ^2^) = 0.086
*S* = 1.00
3143 reflections
268 parameters
H-atom parameters constrained
Δρ_max_ = 0.25 e Å^−3^
Δρ_min_ = −0.13 e Å^−3^

### 

Data collection: *CrystalClear* (Rigaku/MSC, 2008 ▶); cell refinement: *CrystalClear*; data reduction: *CrystalClear*; program(s) used to solve structure: *SHELXS97* (Sheldrick, 2008 ▶); program(s) used to refine structure: *SHELXL97* (Sheldrick, 2008 ▶; molecular graphics: *SHELXTL* (Sheldrick, 2008 ▶; software used to prepare material for publication: *publCIF* (Westrip, 2010 ▶) and *PLATON* (Spek, 2009 ▶).

## Supplementary Material

Crystal structure: contains datablocks I, global. DOI: 10.1107/S1600536810052906/cv5014sup1.cif

Structure factors: contains datablocks I. DOI: 10.1107/S1600536810052906/cv5014Isup2.hkl

Additional supplementary materials:  crystallographic information; 3D view; checkCIF report

## Figures and Tables

**Fig. 1.**
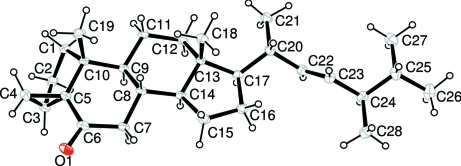
The molecular structure of (I) shown with 30% probability displacement ellipsoids.

## supplementary crystallographic information

### Comment

Many analogues of brassinolide as plant regulator have been isolated from a wide variety of plants, and there has been intense activity in synthesis of these brassinosteroids. We found the same phenomena that our immediates and products were synthesized with impurities by McMorris and Brosa's methods. Herewith we present the crystal structure of the title compound, (I).

In the title molecule, ring A shows an envelope conformation, and atoms C2, C3, C5 and C10 are coplanar with the r.m.s. deviation of 0.018 (1) Å. Atom C1 deviates 0.446 (3) Å from this mean plane, which make a dihedral angle of 68.20 (13) ° with the plane C3/C4/C5. Rings B and C have regular chair conformations, respectively; while ring D has an envelope conformation.

### Experimental

(22*E*,24*R*)-3α,5-Cyclo-5α-ergosta-22-en-6-one was synthesized according to the known method (McMorris *et al.*, 1993) as a powder. Crystals of (I) suitable for structure analysis were obtained by slow evaporation from a mixture of EtOAC and ethanol (volume proportion, 9:1) as colourless prisms.

### Refinement

C-bound H atoms were placed at calculated positions (C—H 0.93–0.98 Å) and constrained to ride on their parent atoms, with*U*_iso_(H_methyl_) = 1.5*U*_eq_(C_methyl_) or *U*_iso_(H_non-methyl_) = 1.2*U*_eq_(C_non-methyl_). Because of negligible anomalous scattering effects, 2359 Friedel pairs were averaged in the refinement. The absolute configuration has been assigned to correspond to the known chiral centres in a precursor molecule, which remained unchanged during the synthesis of (I).

### Crystal data

**Table:**

C_28_H_44_O	*D*_x_ = 1.095 Mg m^−^^3^
*M**_r_* = 396.63	Melting point = 441–442 K
Orthorhombic, *P*2_1_2_1_2_1_	Mo *K*α radiation, λ = 0.71073 Å
Hall symbol: P 2ac 2ab	Cell parameters from 7977 reflections
*a* = 7.6628 (19) Å	θ = 3.3–27.5°
*b* = 10.516 (3) Å	µ = 0.06 mm^−^^1^
*c* = 29.855 (8) Å	*T* = 123 K
*V* = 2405.9 (11) Å^3^	Block, colourless
*Z* = 4	0.42 × 0.36 × 0.34 mm
*F*(000) = 880	

### Data collection

**Table:**

Rigaku AFC10/Saturn724+ diffractometer	2973 reflections with *I* > 2σ(*I*)
Radiation source: Rotating Anode	*R*_int_ = 0.028
graphite	θ_max_ = 27.5°, θ_min_ = 3.3°
Detector resolution: 28.5714 pixels mm^-1^	*h* = −9→8
phi and ω scans	*k* = −12→13
18847 measured reflections	*l* = −38→38
3143 independent reflections	

### Refinement

**Table:**

Refinement on *F*^2^	Primary atom site location: structure-invariant direct methods
Least-squares matrix: full	Secondary atom site location: difference Fourier map
*R*[*F*^2^ > 2σ(*F*^2^)] = 0.038	Hydrogen site location: inferred from neighbouring sites
*wR*(*F*^2^) = 0.086	H-atom parameters constrained
*S* = 1.00	*w* = 1/[σ^2^(*F*_o_^2^) + (0.0381*P*)^2^ + 0.526*P*] where *P* = (*F*_o_^2^ + 2*F*_c_^2^)/3
3143 reflections	(Δ/σ)_max_ < 0.001
268 parameters	Δρ_max_ = 0.25 e Å^−^^3^
0 restraints	Δρ_min_ = −0.13 e Å^−^^3^

### Special details

**Table:**

Geometry. All e.s.d.'s (except the e.s.d. in the dihedral angle between two l.s. planes) are estimated using the full covariance matrix. The cell e.s.d.'s are taken into account individually in the estimation of e.s.d.'s in distances, angles and torsion angles; correlations between e.s.d.'s in cell parameters are only used when they are defined by crystal symmetry. An approximate (isotropic) treatment of cell e.s.d.'s is used for estimating e.s.d.'s involving l.s. planes.
Refinement. Refinement of *F*^2^ against ALL reflections. The weighted *R*-factor *wR* and goodness of fit *S* are based on *F*^2^, conventional *R*-factors *R* are based on *F*, with *F* set to zero for negative *F*^2^. The threshold expression of *F*^2^ > σ(*F*^2^) is used only for calculating *R*-factors(gt) *etc*. and is not relevant to the choice of reflections for refinement. *R*-factors based on *F*^2^ are statistically about twice as large as those based on *F*, and *R*- factors based on ALL data will be even larger.

### Fractional atomic coordinates and isotropic or equivalent isotropic displacement parameters (Å^2^)

**Table:**

	*x*	*y*	*z*	*U*_iso_*/*U*_eq_	
O1	0.30218 (17)	0.98106 (15)	0.72666 (5)	0.0450 (4)	
C1	0.8956 (2)	1.11208 (17)	0.71079 (6)	0.0336 (4)	
H1A	0.9464	1.1250	0.7409	0.040*	
H1B	0.9918	1.1069	0.6887	0.040*	
C2	0.7725 (3)	1.22209 (17)	0.69899 (7)	0.0386 (4)	
H2A	0.8111	1.3020	0.7135	0.046*	
H2B	0.7678	1.2353	0.6662	0.046*	
C3	0.5968 (2)	1.18107 (18)	0.71677 (7)	0.0388 (4)	
H3	0.4893	1.2217	0.7043	0.047*	
C4	0.5881 (3)	1.1216 (2)	0.76191 (6)	0.0456 (5)	
H4A	0.4750	1.1246	0.7779	0.055*	
H4B	0.6920	1.1292	0.7814	0.055*	
C5	0.6013 (2)	1.03587 (18)	0.72191 (6)	0.0318 (4)	
C6	0.4414 (2)	0.96834 (18)	0.70745 (6)	0.0320 (4)	
C7	0.4549 (2)	0.89255 (17)	0.66471 (5)	0.0282 (3)	
H7A	0.4275	0.9492	0.6392	0.034*	
H7B	0.3657	0.8245	0.6653	0.034*	
C8	0.6335 (2)	0.83190 (15)	0.65663 (5)	0.0241 (3)	
H8	0.6507	0.7629	0.6792	0.029*	
C9	0.7817 (2)	0.92938 (15)	0.66207 (5)	0.0236 (3)	
H9	0.7577	1.0002	0.6406	0.028*	
C10	0.7850 (2)	0.98849 (17)	0.70976 (5)	0.0276 (3)	
C11	0.9591 (2)	0.87132 (17)	0.64884 (6)	0.0275 (3)	
H11A	1.0478	0.9397	0.6484	0.033*	
H11B	0.9942	0.8092	0.6721	0.033*	
C12	0.9587 (2)	0.80429 (16)	0.60302 (5)	0.0265 (3)	
H12A	0.9410	0.8683	0.5791	0.032*	
H12B	1.0735	0.7635	0.5981	0.032*	
C13	0.8151 (2)	0.70341 (14)	0.59999 (5)	0.0235 (3)	
C14	0.64205 (19)	0.77281 (15)	0.61000 (5)	0.0237 (3)	
H14	0.6349	0.8450	0.5883	0.028*	
C15	0.5007 (2)	0.67829 (17)	0.59621 (6)	0.0306 (4)	
H15A	0.3908	0.7227	0.5885	0.037*	
H15B	0.4772	0.6165	0.6205	0.037*	
C16	0.5795 (2)	0.61158 (16)	0.55473 (6)	0.0296 (4)	
H16A	0.5175	0.6385	0.5272	0.035*	
H16B	0.5690	0.5181	0.5577	0.035*	
C17	0.7754 (2)	0.65110 (15)	0.55229 (5)	0.0247 (3)	
H17	0.7838	0.7243	0.5310	0.030*	
C18	0.8492 (2)	0.59299 (16)	0.63263 (5)	0.0304 (4)	
H18A	0.9635	0.5553	0.6262	0.046*	
H18B	0.7582	0.5283	0.6290	0.046*	
H18C	0.8476	0.6250	0.6635	0.046*	
C19	0.8522 (2)	0.89395 (19)	0.74489 (5)	0.0372 (4)	
H19A	0.8319	0.9284	0.7749	0.056*	
H19B	0.9774	0.8801	0.7405	0.056*	
H19C	0.7901	0.8130	0.7417	0.056*	
C20	0.8887 (2)	0.54309 (15)	0.53322 (5)	0.0267 (3)	
H20	0.8758	0.4675	0.5533	0.032*	
C21	1.0830 (2)	0.57688 (17)	0.53063 (6)	0.0338 (4)	
H21A	1.1455	0.5099	0.5144	0.051*	
H21B	1.1306	0.5843	0.5610	0.051*	
H21C	1.0972	0.6580	0.5149	0.051*	
C22	0.8252 (2)	0.50642 (15)	0.48723 (5)	0.0286 (4)	
H22	0.8351	0.5683	0.4642	0.034*	
C23	0.7573 (2)	0.39577 (16)	0.47638 (5)	0.0285 (4)	
H23	0.7463	0.3352	0.4998	0.034*	
C24	0.6951 (2)	0.35497 (16)	0.43073 (5)	0.0287 (4)	
H24	0.7161	0.4265	0.4093	0.034*	
C25	0.7985 (3)	0.23792 (17)	0.41424 (6)	0.0355 (4)	
H25	0.7812	0.1680	0.4365	0.043*	
C26	0.7347 (3)	0.1902 (2)	0.36895 (7)	0.0510 (6)	
H26A	0.7421	0.2592	0.3469	0.077*	
H26B	0.6132	0.1620	0.3716	0.077*	
H26C	0.8076	0.1189	0.3592	0.077*	
C27	0.9935 (3)	0.2666 (2)	0.41187 (6)	0.0413 (5)	
H27A	1.0139	0.3359	0.3905	0.062*	
H27B	1.0563	0.1905	0.4020	0.062*	
H27C	1.0354	0.2920	0.4416	0.062*	
C28	0.4989 (3)	0.3298 (2)	0.43273 (6)	0.0406 (4)	
H28A	0.4766	0.2547	0.4513	0.061*	
H28B	0.4545	0.3148	0.4024	0.061*	
H28C	0.4397	0.4036	0.4458	0.061*	

### Atomic displacement parameters (Å^2^)

**Table:**

	*U*^11^	*U*^22^	*U*^33^	*U*^12^	*U*^13^	*U*^23^
O1	0.0238 (7)	0.0577 (9)	0.0534 (8)	0.0054 (7)	0.0097 (6)	−0.0095 (7)
C1	0.0242 (8)	0.0391 (9)	0.0375 (9)	0.0002 (8)	−0.0065 (7)	−0.0092 (8)
C2	0.0339 (10)	0.0321 (9)	0.0498 (11)	0.0007 (8)	−0.0100 (8)	−0.0125 (8)
C3	0.0303 (10)	0.0372 (9)	0.0489 (10)	0.0098 (8)	−0.0078 (8)	−0.0138 (8)
C4	0.0318 (10)	0.0638 (13)	0.0413 (10)	0.0091 (10)	0.0019 (8)	−0.0193 (10)
C5	0.0226 (8)	0.0393 (9)	0.0335 (8)	0.0072 (8)	−0.0004 (7)	−0.0051 (7)
C6	0.0217 (8)	0.0359 (9)	0.0383 (9)	0.0072 (7)	0.0020 (7)	0.0040 (8)
C7	0.0171 (7)	0.0303 (8)	0.0373 (8)	0.0006 (7)	0.0002 (6)	0.0013 (7)
C8	0.0166 (7)	0.0251 (7)	0.0306 (8)	0.0016 (6)	0.0011 (6)	0.0038 (6)
C9	0.0171 (7)	0.0265 (7)	0.0271 (7)	0.0001 (6)	−0.0015 (6)	0.0008 (6)
C10	0.0193 (8)	0.0349 (9)	0.0285 (8)	0.0040 (7)	−0.0027 (6)	−0.0018 (7)
C11	0.0150 (7)	0.0309 (8)	0.0366 (8)	−0.0004 (6)	−0.0016 (6)	−0.0033 (7)
C12	0.0151 (7)	0.0285 (8)	0.0358 (8)	−0.0011 (6)	0.0029 (6)	−0.0034 (7)
C13	0.0187 (7)	0.0227 (7)	0.0289 (7)	0.0012 (6)	−0.0004 (6)	0.0010 (6)
C14	0.0165 (7)	0.0238 (7)	0.0308 (8)	−0.0008 (6)	−0.0012 (6)	0.0035 (6)
C15	0.0196 (8)	0.0313 (9)	0.0408 (9)	−0.0039 (7)	−0.0005 (7)	−0.0010 (7)
C16	0.0233 (8)	0.0276 (8)	0.0379 (8)	−0.0037 (7)	−0.0024 (7)	0.0004 (7)
C17	0.0215 (8)	0.0230 (7)	0.0295 (7)	−0.0011 (6)	−0.0010 (6)	0.0033 (6)
C18	0.0292 (8)	0.0286 (8)	0.0334 (8)	0.0050 (7)	−0.0005 (7)	0.0036 (7)
C19	0.0304 (9)	0.0514 (11)	0.0299 (8)	0.0075 (9)	−0.0035 (7)	0.0041 (8)
C20	0.0267 (8)	0.0235 (7)	0.0299 (8)	0.0007 (7)	0.0008 (6)	0.0018 (6)
C21	0.0271 (9)	0.0346 (9)	0.0397 (9)	0.0015 (8)	0.0027 (7)	−0.0029 (8)
C22	0.0297 (9)	0.0265 (8)	0.0295 (8)	0.0018 (7)	0.0021 (7)	0.0038 (6)
C23	0.0300 (9)	0.0276 (8)	0.0280 (7)	−0.0006 (7)	0.0009 (6)	0.0056 (7)
C24	0.0309 (9)	0.0266 (8)	0.0285 (8)	−0.0059 (7)	−0.0004 (7)	0.0052 (6)
C25	0.0479 (11)	0.0264 (8)	0.0324 (8)	−0.0017 (8)	0.0036 (8)	0.0029 (7)
C26	0.0627 (15)	0.0449 (11)	0.0456 (11)	−0.0063 (11)	0.0038 (10)	−0.0136 (9)
C27	0.0436 (11)	0.0405 (10)	0.0397 (10)	0.0084 (9)	0.0043 (9)	0.0035 (9)
C28	0.0353 (10)	0.0442 (11)	0.0423 (10)	−0.0095 (9)	−0.0026 (8)	0.0002 (9)

### Geometric parameters (Å, °)

**Table:**

O1—C6	1.219 (2)	C15—H15A	0.9900
C1—C2	1.533 (2)	C15—H15B	0.9900
C1—C10	1.552 (2)	C16—C17	1.559 (2)
C1—H1A	0.9900	C16—H16A	0.9900
C1—H1B	0.9900	C16—H16B	0.9900
C2—C3	1.511 (3)	C17—C20	1.539 (2)
C2—H2A	0.9900	C17—H17	1.0000
C2—H2B	0.9900	C18—H18A	0.9800
C3—C4	1.487 (3)	C18—H18B	0.9800
C3—C5	1.535 (3)	C18—H18C	0.9800
C3—H3	1.0000	C19—H19A	0.9800
C4—C5	1.500 (2)	C19—H19B	0.9800
C4—H4A	0.9900	C19—H19C	0.9800
C4—H4B	0.9900	C20—C22	1.507 (2)
C5—C6	1.481 (2)	C20—C21	1.533 (2)
C5—C10	1.536 (2)	C20—H20	1.0000
C6—C7	1.509 (2)	C21—H21A	0.9800
C7—C8	1.528 (2)	C21—H21B	0.9800
C7—H7A	0.9900	C21—H21C	0.9800
C7—H7B	0.9900	C22—C23	1.316 (2)
C8—C14	1.526 (2)	C22—H22	0.9500
C8—C9	1.539 (2)	C23—C24	1.507 (2)
C8—H8	1.0000	C23—H23	0.9500
C9—C11	1.542 (2)	C24—C28	1.526 (3)
C9—C10	1.554 (2)	C24—C25	1.546 (2)
C9—H9	1.0000	C24—H24	1.0000
C10—C19	1.534 (2)	C25—C26	1.522 (2)
C11—C12	1.539 (2)	C25—C27	1.525 (3)
C11—H11A	0.9900	C25—H25	1.0000
C11—H11B	0.9900	C26—H26A	0.9800
C12—C13	1.531 (2)	C26—H26B	0.9800
C12—H12A	0.9900	C26—H26C	0.9800
C12—H12B	0.9900	C27—H27A	0.9800
C13—C18	1.538 (2)	C27—H27B	0.9800
C13—C14	1.543 (2)	C27—H27C	0.9800
C13—C17	1.558 (2)	C28—H28A	0.9800
C14—C15	1.527 (2)	C28—H28B	0.9800
C14—H14	1.0000	C28—H28C	0.9800
C15—C16	1.546 (2)		
			
C2—C1—C10	106.93 (13)	C13—C14—H14	106.3
C2—C1—H1A	110.3	C14—C15—C16	103.54 (13)
C10—C1—H1A	110.3	C14—C15—H15A	111.1
C2—C1—H1B	110.3	C16—C15—H15A	111.1
C10—C1—H1B	110.3	C14—C15—H15B	111.1
H1A—C1—H1B	108.6	C16—C15—H15B	111.1
C3—C2—C1	104.63 (15)	H15A—C15—H15B	109.0
C3—C2—H2A	110.8	C15—C16—C17	107.05 (13)
C1—C2—H2A	110.8	C15—C16—H16A	110.3
C3—C2—H2B	110.8	C17—C16—H16A	110.3
C1—C2—H2B	110.8	C15—C16—H16B	110.3
H2A—C2—H2B	108.9	C17—C16—H16B	110.3
C4—C3—C2	118.56 (16)	H16A—C16—H16B	108.6
C4—C3—C5	59.46 (13)	C20—C17—C13	119.25 (13)
C2—C3—C5	107.35 (15)	C20—C17—C16	111.34 (13)
C4—C3—H3	118.8	C13—C17—C16	103.83 (12)
C2—C3—H3	118.8	C20—C17—H17	107.3
C5—C3—H3	118.8	C13—C17—H17	107.3
C3—C4—C5	61.86 (12)	C16—C17—H17	107.3
C3—C4—H4A	117.6	C13—C18—H18A	109.5
C5—C4—H4A	117.6	C13—C18—H18B	109.5
C3—C4—H4B	117.6	H18A—C18—H18B	109.5
C5—C4—H4B	117.6	C13—C18—H18C	109.5
H4A—C4—H4B	114.7	H18A—C18—H18C	109.5
C6—C5—C4	117.68 (15)	H18B—C18—H18C	109.5
C6—C5—C3	115.35 (15)	C10—C19—H19A	109.5
C4—C5—C3	58.68 (13)	C10—C19—H19B	109.5
C6—C5—C10	122.22 (14)	H19A—C19—H19B	109.5
C4—C5—C10	116.48 (14)	C10—C19—H19C	109.5
C3—C5—C10	108.69 (15)	H19A—C19—H19C	109.5
O1—C6—C5	122.30 (17)	H19B—C19—H19C	109.5
O1—C6—C7	121.09 (16)	C22—C20—C21	109.13 (14)
C5—C6—C7	116.34 (14)	C22—C20—C17	110.07 (13)
C6—C7—C8	114.52 (13)	C21—C20—C17	113.28 (13)
C6—C7—H7A	108.6	C22—C20—H20	108.1
C8—C7—H7A	108.6	C21—C20—H20	108.1
C6—C7—H7B	108.6	C17—C20—H20	108.1
C8—C7—H7B	108.6	C20—C21—H21A	109.5
H7A—C7—H7B	107.6	C20—C21—H21B	109.5
C14—C8—C7	110.61 (12)	H21A—C21—H21B	109.5
C14—C8—C9	109.61 (12)	C20—C21—H21C	109.5
C7—C8—C9	111.50 (13)	H21A—C21—H21C	109.5
C14—C8—H8	108.3	H21B—C21—H21C	109.5
C7—C8—H8	108.3	C23—C22—C20	125.33 (14)
C9—C8—H8	108.3	C23—C22—H22	117.3
C8—C9—C11	111.11 (12)	C20—C22—H22	117.3
C8—C9—C10	112.03 (12)	C22—C23—C24	126.84 (14)
C11—C9—C10	112.28 (12)	C22—C23—H23	116.6
C8—C9—H9	107.0	C24—C23—H23	116.6
C11—C9—H9	107.0	C23—C24—C28	109.02 (14)
C10—C9—H9	107.0	C23—C24—C25	110.59 (14)
C19—C10—C5	110.86 (14)	C28—C24—C25	112.30 (15)
C19—C10—C1	110.24 (13)	C23—C24—H24	108.3
C5—C10—C1	102.93 (13)	C28—C24—H24	108.3
C19—C10—C9	111.87 (14)	C25—C24—H24	108.3
C5—C10—C9	109.40 (12)	C26—C25—C27	109.82 (16)
C1—C10—C9	111.20 (13)	C26—C25—C24	112.33 (16)
C12—C11—C9	114.02 (13)	C27—C25—C24	111.04 (15)
C12—C11—H11A	108.7	C26—C25—H25	107.8
C9—C11—H11A	108.7	C27—C25—H25	107.8
C12—C11—H11B	108.7	C24—C25—H25	107.8
C9—C11—H11B	108.7	C25—C26—H26A	109.5
H11A—C11—H11B	107.6	C25—C26—H26B	109.5
C13—C12—C11	111.80 (13)	H26A—C26—H26B	109.5
C13—C12—H12A	109.3	C25—C26—H26C	109.5
C11—C12—H12A	109.3	H26A—C26—H26C	109.5
C13—C12—H12B	109.3	H26B—C26—H26C	109.5
C11—C12—H12B	109.3	C25—C27—H27A	109.5
H12A—C12—H12B	107.9	C25—C27—H27B	109.5
C12—C13—C18	111.35 (13)	H27A—C27—H27B	109.5
C12—C13—C14	106.13 (12)	C25—C27—H27C	109.5
C18—C13—C14	112.40 (12)	H27A—C27—H27C	109.5
C12—C13—C17	116.03 (12)	H27B—C27—H27C	109.5
C18—C13—C17	110.25 (12)	C24—C28—H28A	109.5
C14—C13—C17	100.14 (12)	C24—C28—H28B	109.5
C8—C14—C15	118.71 (13)	H28A—C28—H28B	109.5
C8—C14—C13	113.96 (12)	C24—C28—H28C	109.5
C15—C14—C13	104.43 (12)	H28A—C28—H28C	109.5
C8—C14—H14	106.3	H28B—C28—H28C	109.5
C15—C14—H14	106.3		
			
C10—C1—C2—C3	31.09 (18)	C11—C9—C10—C1	70.92 (16)
C1—C2—C3—C4	43.3 (2)	C8—C9—C11—C12	50.03 (18)
C1—C2—C3—C5	−20.85 (19)	C10—C9—C11—C12	176.38 (13)
C2—C3—C4—C5	−94.19 (18)	C9—C11—C12—C13	−54.27 (18)
C3—C4—C5—C6	−104.28 (18)	C11—C12—C13—C18	−65.93 (17)
C3—C4—C5—C10	96.66 (18)	C11—C12—C13—C14	56.69 (16)
C4—C3—C5—C6	108.27 (17)	C11—C12—C13—C17	166.88 (13)
C2—C3—C5—C6	−138.33 (15)	C7—C8—C14—C15	−53.32 (18)
C2—C3—C5—C4	113.41 (17)	C9—C8—C14—C15	−176.67 (13)
C4—C3—C5—C10	−110.19 (16)	C7—C8—C14—C13	−177.00 (13)
C2—C3—C5—C10	3.2 (2)	C9—C8—C14—C13	59.65 (16)
C4—C5—C6—O1	0.4 (3)	C12—C13—C14—C8	−61.60 (15)
C3—C5—C6—O1	−66.0 (2)	C18—C13—C14—C8	60.35 (17)
C10—C5—C6—O1	158.13 (18)	C17—C13—C14—C8	177.35 (12)
C4—C5—C6—C7	174.52 (15)	C12—C13—C14—C15	167.31 (13)
C3—C5—C6—C7	108.16 (18)	C18—C13—C14—C15	−70.75 (15)
C10—C5—C6—C7	−27.7 (2)	C17—C13—C14—C15	46.26 (14)
O1—C6—C7—C8	−152.17 (17)	C8—C14—C15—C16	−164.04 (13)
C5—C6—C7—C8	33.6 (2)	C13—C14—C15—C16	−35.79 (15)
C6—C7—C8—C14	−172.34 (13)	C14—C15—C16—C17	11.02 (16)
C6—C7—C8—C9	−50.10 (18)	C12—C13—C17—C20	83.68 (17)
C14—C8—C9—C11	−50.93 (16)	C18—C13—C17—C20	−44.06 (18)
C7—C8—C9—C11	−173.74 (13)	C14—C13—C17—C20	−162.65 (13)
C14—C8—C9—C10	−177.42 (12)	C12—C13—C17—C16	−151.75 (13)
C7—C8—C9—C10	59.77 (16)	C18—C13—C17—C16	80.52 (15)
C6—C5—C10—C19	−88.33 (19)	C14—C13—C17—C16	−38.08 (14)
C4—C5—C10—C19	69.7 (2)	C15—C16—C17—C20	146.76 (13)
C3—C5—C10—C19	133.29 (16)	C15—C16—C17—C13	17.23 (16)
C6—C5—C10—C1	153.80 (16)	C13—C17—C20—C22	179.04 (13)
C4—C5—C10—C1	−48.18 (19)	C16—C17—C20—C22	58.18 (17)
C3—C5—C10—C1	15.43 (17)	C13—C17—C20—C21	−58.47 (18)
C6—C5—C10—C9	35.5 (2)	C16—C17—C20—C21	−179.34 (13)
C4—C5—C10—C9	−166.48 (15)	C21—C20—C22—C23	120.14 (18)
C3—C5—C10—C9	−102.87 (15)	C17—C20—C22—C23	−114.96 (19)
C2—C1—C10—C19	−146.85 (15)	C20—C22—C23—C24	−179.02 (15)
C2—C1—C10—C5	−28.55 (17)	C22—C23—C24—C28	−116.44 (19)
C2—C1—C10—C9	88.48 (16)	C22—C23—C24—C25	119.62 (19)
C8—C9—C10—C19	73.02 (17)	C23—C24—C25—C26	177.68 (15)
C11—C9—C10—C19	−52.83 (18)	C28—C24—C25—C26	55.6 (2)
C8—C9—C10—C5	−50.22 (17)	C23—C24—C25—C27	−58.90 (18)
C11—C9—C10—C5	−176.07 (14)	C28—C24—C25—C27	179.07 (15)
C8—C9—C10—C1	−163.23 (12)		
